# The microcirculatory characteristics of the heart and lung meridians: Study protocol clinical trial (SPIRIT Compliant)

**DOI:** 10.1097/MD.0000000000019594

**Published:** 2020-04-03

**Authors:** Hantong Hu, Yongliang Jiang, Xiaoyu Li, Jiali Lou, Yajun Zhang, Xiaofen He, Junfan Fang, Yuanyuan Wu, Xiaomei Shao, Jianqiao Fang

**Affiliations:** a The Third Affiliated Hospital of Zhejiang Chinese Medical University, Hangzhou City, Zhejiang Province; b Department of Neurobiology and Acupuncture Research, The Third Clinical Medical College, Zhejiang Chinese Medical University, Key Laboratory of Acupuncture and Neurology of Zhejiang Province, Hangzhou, China.

**Keywords:** chronic stable angina pectoris, laser doppler flowmetry, microcirculation, site specificity, trials

## Abstract

**Introduction:**

The aim of the present study is to compare the microcirculatory difference of different meridians by using laser doppler flowmetry and investigate the specificity for the meridian-visceral association and site-to-site association between 2 specific meridians.

**Methods and analysis:**

The Lung and Heart meridians are chosen as 2 specific studied meridians. 120 participants will be enrolled and divided into the healthy control group, chronic stable angina pectoris group and healthy intervention group. Laser doppler flowmetry will be used to assess the blood perfusion of the Heart and Lung meridians. The specificity for the meridian-visceral association will be investigated by comparing the microcirculatory difference between the Heart and Lung meridians in the healthy control group and chronic stable angina pectoris group. Besides, participants in the healthy intervention group will receive 2 sessions of moxibustion in the Heart meridian and Lung meridian, respectively, to explore the specificity for the site-to-site association on the body surface. Primary outcomes will be blood flow curve and blood perfusion units of relevant sites along the Heart and Lung meridians. Statistical analysis will be conducted by third party statisticians.

**Ethics and dissemination:**

Ethics approval (approval No: ZSLL-KY-2019-001A-01) has been obtained from the Ethics Committee of the Third Affiliated Hospital of Zhejiang Chinese Medical University. The study findings will be disseminated through presentation at peer-reviewed medical journals.

**Trial registration:**

ClinicalTrials.gov NCT04244812.

## 1 Introduction

In recent years, acupuncture has received increasing attention in many Western countries. Convincing evidence based on meta-analyses has proved that acupuncture is effective for treating a wide range of diseases, such as pain conditions,^[1,2]^ respiratory diseases,^[3,4]^ cardiovascular disorders^[5,6]^ and digestive diseases.^[7,8]^ Although acupuncture gains increasing acceptance, as the guidance of almost all acupuncture clinical practices for thousands of years,^[9]^ the existence of meridian systems and meridian theory have been questioned.^[10,11]^

According to the theories of acupuncture and traditional Chinese medicine, meridians distribute on the surface of the whole body vertically and horizontally, integrating the surface of the body with internal organs, thus transforming the whole body into one entire organ.^[12]^ That is to say, the essence of the meridian theory and meridian systems mainly manifests its summaries concerning the fundamental rules for correlation and specificity of different sites of the body. After many years of effort, although the physical structure of meridians has not been found, the biological characteristics of meridians has been confirmed,^[9]^ which could be used as the entry point for meridian studies. However, the majority of the existing studies involve lots of subjective assessments. Moreover, few studies have investigated the site specificity between 2 specific meridians.

Therefore, the present study adopts laser doppler flowmetry (LDF) to assess the microcirculatory characteristics of meridians, which is an established approach for monitoring blood flux in the microcirculation^[13]^ with advantages of non-invasiveness, measurement of rapid response, ease of application, continuity, and tissue specificity.^[14]^

The aim of the present study is to compare the difference in the microcirculatory characteristics of 2 specific meridians. Specifically, the specificity for the meridian-visceral association and site-to-site association between 2 specific meridians will be investigated. The Heart and Lung meridians are chosen as the 2 studied meridians. Thus, patients with chronic stable angina pectoris (CSAP) and healthy adults will be included. The findings of this study will provide evidence for the biological basis of meridians and bring important reference for the acupoint selection in acupuncture clinical practice.

## 2 Methods and design

### 2.1 Study design

**Figure 1:**
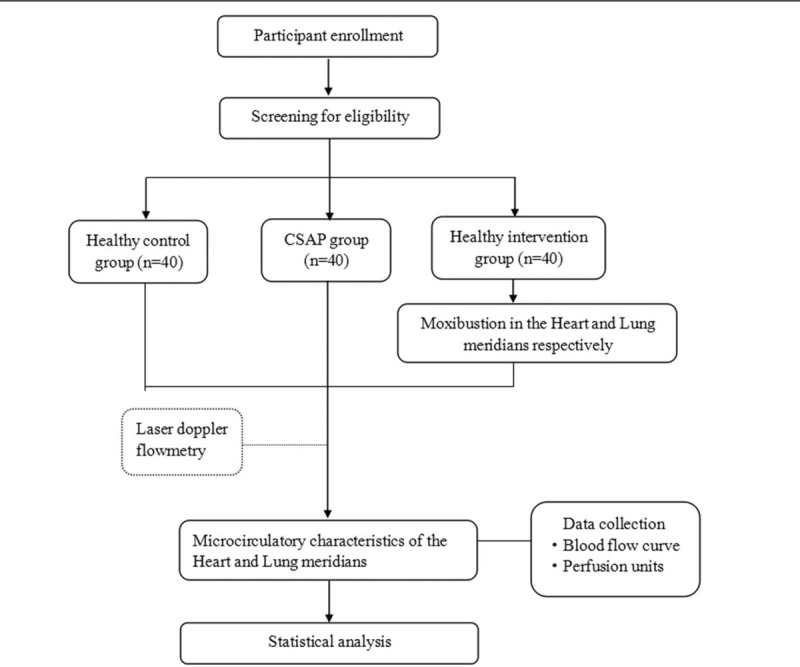
Study design flowchart. CSAP = chronic stable angina pectoris.

We will conduct a prospective and clinical controlled trial. All participants will be divided into the healthy control group, CSAP group and healthy intervention group. The standard protocol items including Recommendations for Interventional Trials and the Standards for Reporting Interventions in Clinical Trials of Acupuncture guidelines will be followed during the development of this trial protocol. The study design flowchart is shown in Figure 1 and the schedule of enrolment, interventions and assessments is displayed in Table 1.

**Table 1:**
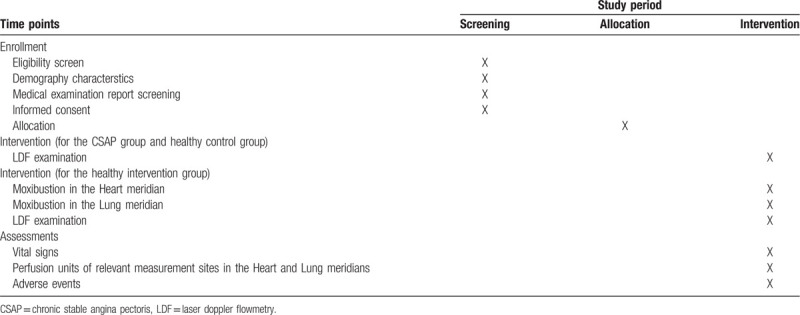
Trial schedule of enrollment, intervention and assessments.

### 2.2 Sample size estimation

This trial is a meridian study that uses a modern technique to assess the biological characteristics of meridians. Compared with general clinical trials, there is no unified standard for the sample size estimation. Based on similar meridian studies,^[15–17]^ a total of 120 participants will be included and divided into the healthy control group, CSAP group and healthy intervention group, with 40 subjects in each group respectively.

### 2.3 Participants

The subjects of this study include healthy adults and CSAP patients.

#### 2.3.1 Inclusion criteria

##### 2.3.1.1 Inclusion criteria for healthy adults

(1) Healthy adults should provide a recent medical examination report to confirm they have not any cardiovascular, respiratory, digestive, urinary, hematological, endocrine, and neurological disease;
(2) Age ≥20 years, male or female;
(3) Participants have clear consciousness and could communicate with others normally;
(4) Participants could understand the full study protocol and written informed consent is signed.

##### 2.3.1.2 Inclusion criteria for the CSAP patients

(1) Patients should meet the diagnostic criteria of coronary heart disease, which includes the following items: 1) confirmed old myocardial infarction, or a history of percutaneous coronary intervention, or coronary artery bypass grafting; 2) 50% or more luminal stenosis in at least one coronary artery or major branch segment confirmed by coronary angiogram or CT angiography; 3) myocardial ischemia indicted by exercise stress radionuclide myocardial imaging; 4) treadmill exercise testing is positive;
(2) Patients should meet the diagnostic criteria of CSAP and the Canadian Cardiovascular Society classification for CSAP is level II or III;
(3) The medical history of CSAP is more than 3 months with attacks occurring at least twice weekly in the last month;
(4) 35≤age≤75 years, male or female;
(5) Patients have clear consciousness and could communicate with others normally;
(6) Patients could understand the full study protocol and written informed consent is signed.

#### 2.3.2 Exclusion criteria

##### 2.3.2.1 Exclusion criteria of the healthy adults

(1) Participants have mental illness, severe depression, alcohol dependence or history of drug abuse;
(2) Pregnant or lactating participants;
(3) Participants are participating in other trials.

##### 2.3.2.2 Exclusion criteria for the CSAP patients

(1) Patients have acute coronary syndrome and severe arrhythmias;
(2) Patients’ chest pain is caused by valvular heart disease, hypertrophic cardiomyopathy and dilated cardiomyopathy;
(3) Patients’ chest pain is caused by non-cardiac disease;
(4) Patients have concomitant lung diseases, such as obstructive pulmonary disease;
(5) Patients have serious concomitant conditions and fail to treat them effectively, such as diseases of the digestive, urinary, respiratory, haematological, and nervous system;
(6) Patients have mental illness, severe depression, alcohol dependence or history of drug abuse;
(7) Pregnant or lactating patients;
(8) Patients are participating in other trials.

### 2.4 Recruitment procedures

This trial will be performed at the Third Affiliated Hospital of Zhejiang Chinese Medical University. A total of 120 participants will be recruited through posters and networks. All participants will be informed of the purpose, contents, benefits and potential risks of the study. And the written informed consent will be signed.

### 2.5 Blinding

This is an open-labeled controlled trial. The participants, manipulators and outcome assessors will not be blinded. In the data analysis stage, statistical analysis will be conducted by third party statisticians who are blinded to the study protocol.

### 2.6 Experimental procedures

All participants in 3 groups will receive examination of LDF. Besides, participants in the healthy control group will receive moxibustion intervention in the Heart and Lung meridians successively.

To minimize the interference effect induced by confounding factors, all LDF examinations will be performed in a quiet experimental room, controlled for temperature and humidity (24°C ± 1°C; relative humidity 30%–40%), in the morning at about the same time of day. All subjects will be refrained from consuming coffee, tea, alcohol, or smoking cigarettes on the examination day. Exercise and food will also be restricted at least 1 hour before experiment.

#### 2.6.1 Procedures for LDF examination and moxibustion

A four-channel LDF (PeriFlux System 5000, Sweden) will be used to measure the microcirculatory characteristics of the Heart and Lung meridians, which could monitor 4 measurement sites simultaneously. The participants will be asked to stabilize for 15 minutes in a supine position in the experimental room before LDF examination. They will be informed to keep silent and normal breath and avoid limb movement during the whole measurement period. The probes will be placed at corresponding measurement sites. Blood flow curve and microcirculatory flux, expressed as blood perfusion units (PU), will be assessed.

**Figure 2:**
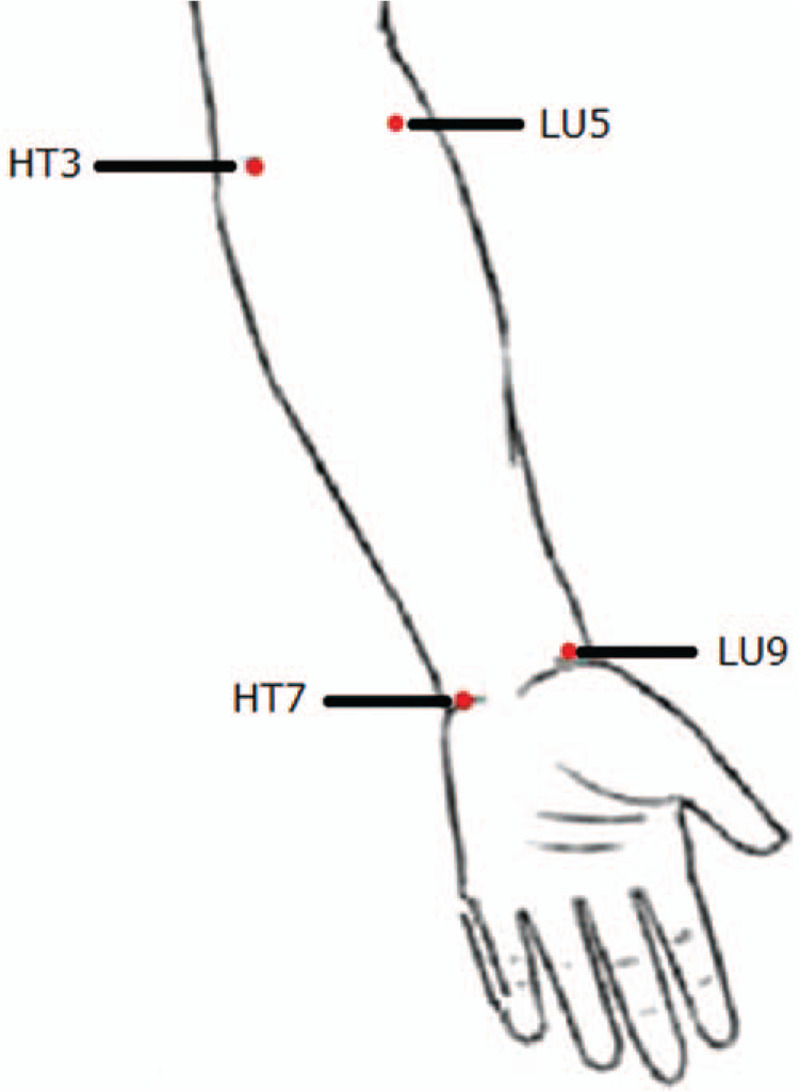
The LDF measurement sites of the healthy control group and CSAP group. CSAP = chronic stable angina pectoris, LDF = laser doppler flowmetry.

**Figure 3:**
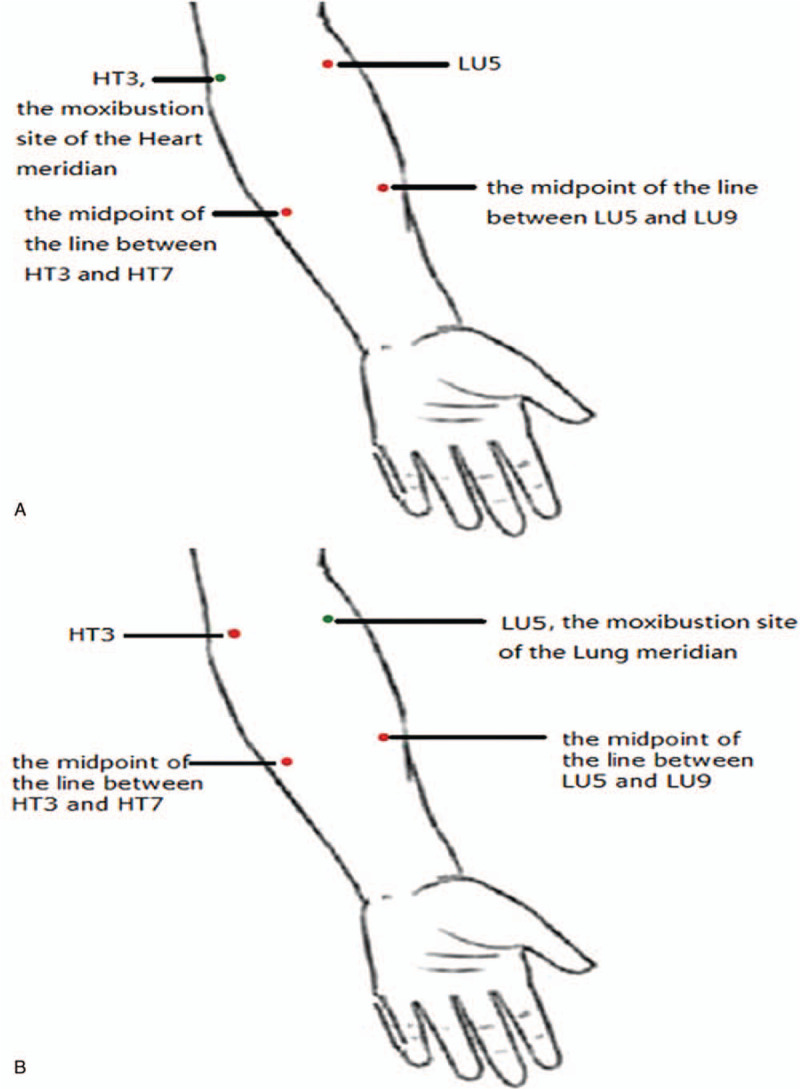
The intervention procedure of the healthy intervention group. (A) Moxibustion site of the Heart meridian and LDF measurement sites of the Heart and Lung meridians; (B) Moxibustion site of the Lung meridian and LDF measurement sites of the Heart and Lung meridians. CSAP = chronic stable angina pectoris, LDF = laser doppler flowmetry.

(1) Healthy control group and CSAP group
  The probes will be left at 4 measurement sites (Fig. 2), which include “Shenmen” (HT7) and “Shaohai” (HT3) of the Heart meridian, “Taiyuan” (LU9) and “Chize” (LU5) of the Lung meridian. The blood flow curve and PU will be recorded for 5 minutes.
(2) Healthy intervention group
  Two sessions of moxibustion will be performed in the Heart meridian and Lung meridian, respectively. The washout period between the two sessions is at least one day to avoid inter-experiment interference. The intervention procedures and LDF measurement sites are shown in Figure 3.

(1) Intervention in the Heart meridian: By igniting the moxa stick and inserting it into a homemade moxibustion holder to adjust appropriate angle and height, moxibustion will be performed at “Shaohai” (HT3) of the Heart meridian for 15 minutes. During moxibustion, LDF probes will be placed in three measurement sites, which include the midpoint of the Heart meridian along the left forearm (ie, midpoint of between HT3 and HT7), “Chize (LU5)” of the Lung meridian, and the midpoint of the Lung meridian along the left forearm(ie, midpoint of between LU5 and LU9). The intervention procedure and LDF measurement sites are shown in Figure 3 (A). The measurement time points include 5 minutes before moxibustion, 15 minutes during moxibustion and 5 minutes after removal of moxibustion.
(2) 2)Intervention in the Lung meridian: The moxibustion acupoint is “Chize” (LU5) of the Lung meridian. The LDF examination, moxibusition procedure and measurement time points are the same as 1). During moxibustion, the LDF probes will detect the blood perfusion in three measurement sites, which include the midpoint of the Lung meridian along the left forearm(i.e. midpoint between LU5 and LU9), “Shaohai” (HT3) of the Heart meridian, and the midpoint of the Heart meridian along the left forearm (i.e. midpoint between HT3 and HT7. The intervention procedure and LDF measurement sites are shown in Figure 3(B).

#### 2.6.2 Locations of the acupoints and LDF measurement sites

The locations of the acupoints and LDF measurement sites of the Heart and Lung meridians are displayed in Table 2.

**Table 2:**
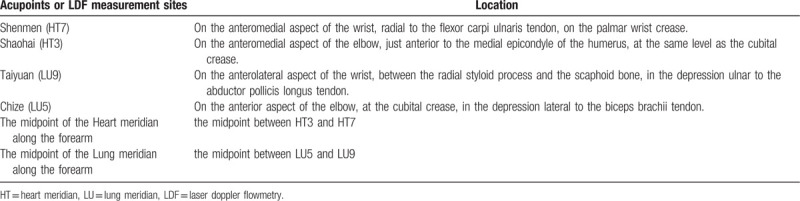
Location of acupoints and LDF measurement sites of the heart and lung meridians.

#### 2.6.3 Concomitant treatments

During the study period, all the participants in the CSAP group will maintain their previous treatment regimen. If additional medications or other treatments are used during the study period due to any reasons, the details (eg, the name, administration time and dosage of the medication) should be documented.

Besides, participants in the healthy control group and healthy intervention group should not take any medications during the full study period. If drugs or other treatments are adopted due to sudden diseases, researchers will evaluate whether they should be withdrawn from the study.

### 2.7 Outcome measures

Primary outcomes include

(1) blood flow curve and
(2) blood PU of relevant sites along the Heart and Lung meridians.

### 2.8 Safety assessment

Adverse events should be recorded and assessed by the investigators during the trial. If serious adverse events occur, the researchers should report them to the principal investigator and ethics committee immediately.

### 2.9 Quality control

Prior to the trial, this protocol has been modified according to suggestions from experienced acupuncturists. All researchers will undergo a series of training sessions on the purpose and standard operating procedures of the trial. Monitors will verify case report forms as well as the LDF examination procedure during the study. Dropouts and withdrawals including the reasons will be documented in details throughout the trial.

### 2.10 Data collection and statistical analysis

Statistical analysis will be performed using the statistical software package SPSS 17.0 for Windows (SPSS Inc., Chicago, IL) by third party statisticians who does not participant in this trial. Numeric data with normal distribution will be expressed as mean ± standard deviations, whereas data with skewed distribution as median with 95% confidence intervals. Categorical data will be shown as counts and percentages.

Repeated measures ANOVA will be used to assess change in continuous variables before and after intervention. The paired samples *t*-test will be used to compare the changes within the groups, whereas the independent samples t-test will be employed for comparisons between the groups. Within-group/between-group comparison regarding data with skewed distribution is assessed using non-parametric test. Between-group differences for baseline dichotomous variables, such as gender and ages, will be tested using the Chi-square test. A *P* value of less than .05 will be considered statistically significant.

### 2.11 Patient and public involvement

Patients or the public are not involved in the design, or conduct, or reporting, or dissemination plans of our research.

### 2.12 Ethics and dissemination

The study is planned in accordance with the Declaration of Helsinki. Ethics approval (approval No: ZSLL-KY-2019-001A-01) has been obtained from the Ethics Committee of the Third Affiliated Hospital of Zhejiang Chinese Medical University. All the participants will be fully informed about this trial and given enough time to inquire about details involving the purpose, contents, benefits and potential risks of the study. Informed consent will be signed if they agree to participate.

The trial has been registered at Clinical Trial Registry with the identification code NCT04244812. The study findings will be disseminated through presentation at peer-reviewed journals.

## 3 Discussion

By far, the biological characteristics of meridians has been investigated. Over the last few decades, results from many studies have suggested distinctive biophysical features of the meridians, such as high electrical conductance,^[18–20]^ nitric oxide levels,^[21,22]^ high transcutaneous CO_2_ emissions,^[23]^ acupuncture sensation patterns,^[24]^ and possible relationships with connective tissue.^[25,26]^ Nevertheless, the scientific evidence on the microcirculatory characteristics of meridians still needs further explosion by using modern scientific techniques.

In addition, the topic of acupoint/site specificity has received increasing attention in acupuncture research. According to the classical acupuncture theory, the effects of acupuncture are site specific, which are also supported by results from modern studies. For examples, Gao et al^[27]^ demonstrated that acupuncture stimulation of the ear had a more significant inhibitory effect on mean arterial pressure and heart rate than that of the lower leg and forearm. Minagawa et al^[28]^ also reported the site-specific, organ-selective effect of an epifascial acupuncture stimulation. Significant neuroimaging evidence of acupoint specificity of the vision-related acupoints was provided by Cho et al.^[29]^ However, no consensus has yet been reached on the existence of acupoint/site specificity.^[12]^

Therefore, we have designed an ongoing clinical trial to compare the microcirculatory characteristics of 2 specific meridians by using an objective assessment tool and verify the specificity between 2 specific meridians. The highlights and strengths of this study are presented as below.

First, to the best of our knowledge, this trial is the first study to compare the difference in microcirculatory characteristics of 2 specific meridians by using LDF. Previous studies^[30–32]^ have proved that LDF is a well-established technique to monitor microcirculation flux in acupuncture-related trials due to its non-invasiveness and real-time capability of measurements.

Second, our study design presents different comparisons to verify the specificity for the meridian-visceral association and site-to-site association between 2 specific meridians, which is a highlight. The theory of meridian-viscera association emphasizes the diagnostic and therapeutic values based on the mutual relation in physiology and the interaction effect in pathology between meridians and viscera. In details, visceral physiological functions and pathological changes will manifest in the corresponding meridians or the acupoints^[33]^ and the visceral disorders can be treated with the involved meridian and acupoints.^[34]^ A number of modern studies have confirmed the relatively specific relationship between meridians and viscera, and the modulating effect of acupoints/meridians for the visceral function^[35–37]^ On 1 hand, we hypothesize that the change in microcirculatory characteristics of the Heart meridian between the healthy control group and CSAP group is more significant than that of the Lung meridian. Thus, the specificity for the meridian-visceral association will be investigated by comparing the microcirculatory difference between the Heart and Lung meridians in the healthy control group and CSAP group. We present such a comparison (healthy control group vs CSAP group) to verify the sensitization phenomena of meridians under pathological circumstances as well as the specificity of meridian-visceral association.

On the other hand, we hypothesize that the change in microcirculatory characteristics in relevant sites of the stimulated meridian is more significant than that of the non-stimulated meridian in the healthy control group. Thus, the specificity for the site-to-site association will be explored by comparing the microcirculatory change between the Heart and Lung meridians by performing moxibustion in the Heart meridian and Lung meridian, respectively. It should be noted that moxibustion intervention is applied only in the healthy control group rather than the CSAP group. By such a study design and comparison, we attempt to confirm whether meridians are not only sensitized in pathological conditions but also activated in physiological state, which is an important issue to be solved in meridian researches.

To summary, based on rigorous experimental designs, the findings concerning the specificity between two specific meridians could contribute to the selection of appropriate acupoints and help optimize the therapeutic effect of acupuncture in clinical practice. It could bring important references to the study of meridian theory in modern researches.

Despite the highlights and strengths of this study, several limitations have to be addressed. First, the sample size is small. But as a pilot trial, the results of this study will provide evidence for the feasibility of this trial design as well as basic data for similar meridian studies in a large-scale study. It is expected to guide the design of a full-scale trial in the future. Second, the results should be interpreted in caution. A number of factors which can influence adequate interpretation of data must be considered.

## 4 Conclusions

In conclusion, this protocol describes an ongoing clinical trial to compare the microcirculatory characteristics of different meridians by using LDF. Specifically, different comparison groups are designed to verify the specificity for the meridian-visceral association and site-to-site association between two specific meridians. The results of the meridian specificity will provide high-quality evidence for a better acupuncture prescription selection in clinical practice so as to improve the therapeutic effect of acupuncture.

## Author contributions

Jianqiao Fang is responsible for this study. Yongliang Jiang and Hantong Hu designed the trial protocol and drafted the manuscript. Yuanyuan Wu, Junfang Fang and Xiaofen He planned a data analysis solution. Xiaoyu Li, Jiali Lou, Xiaomei Shao and Yajun Zhang participated in recruitment, LDF examination and data collection of participants. All the authors have read, revised and approved this version of the manuscript.

## References

[1] Yuan QL Wang P Liu L Acupuncture for musculoskeletal pain: a meta-analysis and meta-regression of sham-controlled randomized clinical trials. Sci Rep 2016;6:30675. 27471137 10.1038/srep30675 PMC4965798

[2] Vickers AJ Vertosick EA Lewith G Acupuncture for chronic pain: update of an individual patient data meta-analysis. J Pain 2018;19:455–74. 29198932 10.1016/j.jpain.2017.11.005 PMC5927830

[3] Su L Meng L Chen R Acupoint application for asthma therapy in adults: a systematic review and meta-analysis of randomized controlled trials. Forsch Komplementmed 2016;23:16–21. 10.1159/000443813 26978427

[4] Hsieh PC Yang MC Wu YK Acupuncture therapy improves health-related quality of life in patients with chronic obstructive pulmonary disease: a systematic review and meta-analysis. Complement Ther Clin Pract 2019;35:208–18. 31003660 10.1016/j.ctcp.2019.02.016

[5] Yang M Sun M Du T The efficacy of acupuncture for stable angina pectoris: a systematic review and meta-analysis. Eur J Prev Cardiol 2019;17: 2047487319876761. 10.1177/2047487319876761 31529993

[6] Liu Y Meng HY Khurwolah MR Acupuncture therapy for the treatment of stable angina pectoris: An updated meta-analysis of randomized controlled trials. Complement Ther Clin Pract 2019;34:247–53. 30712735 10.1016/j.ctcp.2018.12.012

[7] Lan L Zeng F Liu GJ Acupuncture for functional dyspepsia. Cochrane Database Syst Rev 2014;13:Cd008487. 10.1002/14651858.CD008487.pub2 PMC10558101 25306866

[8] Zheng H Chen R Comparison between the effects of acupuncture relative to other controls on irritable bowel syndrome. a meta-analysis. Pain Res Manag 2019;2019:2871505. 31814859 10.1155/2019/2871505 PMC6877908

[9] Wang GJ Ayati MH Zhang WB Meridian studies in China: a systematic review. J Acupunct Meridian Stud 2010;3:1–9. 20633509 10.1016/S2005-2901(10)60001-5

[10] Litscher G Infrared thermography fails to visualize stimulation-induced meridian-like structures. Biomed Eng Online 2005;4:38. 15958163 10.1186/1475-925X-4-38 PMC1180459

[11] Haake M Muller HH Schade-Brittinger C German Acupuncture Trials (GERAC) for chronic low back pain: randomized, multicenter, blinded, parallel-group trial with 3 groups. Arch Intern Med 2007;167:1892–8. 17893311 10.1001/archinte.167.17.1892

[12] Yang J Zeng F Feng Y A PET-CT study on the specificity of acupoints through acupuncture treatment in migraine patients. BMC Complement Altern Med 2012;12:123. 22894176 10.1186/1472-6882-12-123 PMC3480944

[13] Ovadia-Tirosh Z Kornowski R Walden R An integrated noninvasive system for monitoring the microcirculatory effects of vasoactive drugs: an experimental study. Microvasc Res 1997;53:14–21. 9056472 10.1006/mvre.1996.1982

[14] Kouadio AA Jordana F Koffi NJ The use of laser Doppler flowmetry to evaluate oral soft tissue blood flow in humans: a review. Arch Oral Biol 2018;86:58–71. 29182953 10.1016/j.archoralbio.2017.11.009

[15] Xu JS Pan XH Hu XL Comparison between governor meridian and Its bilateral control points in microcirculatory blood perfusion in 53 volunteer subjects. Zhen Ci Yan Jiu 2008;33:321–5. 19097504

[16] Zheng SX Xu JS Pan XH Comparison of microcirculatory blood perfusion between acupoints of the stomach meridian and their bilateral control points and changes of blood flow after electroacupuncture in 21 volunteer subjects. Zhen Ci Yan Jiu 2012;1:53–8. 22574570

[17] Hsiu H Huang SM Chao PT Microcirculatory characteristics of acupuncture points obtained by laser Doppler flowmetry. Physiol Meas 2007;28:N77–86. 17906382 10.1088/0967-3334/28/10/N01

[18] Ahn AC Colbert AP Anderson BJ Electrical properties of acupuncture points and meridians: a systematic review. Bioelectromagnetics 2008;29:245–56. 18240287 10.1002/bem.20403

[19] Ahn AC Park M Shaw JR Electrical impedance of acupuncture meridians: the relevance of subcutaneous collagenous bands. PloS one 2010;5:e11907. 20689594 10.1371/journal.pone.0011907 PMC2912845

[20] Gow BJ Cheng JL Baikie ID Electrical potential of acupuncture points: use of a noncontact scanning Kelvin probe. Evid Based Complement Alternat Med 2012;2012:632838. 23320033 10.1155/2012/632838 PMC3541002

[21] Ha Y Kim M Nah J Measurements of location-dependent nitric oxide levels on skin surface in relation to acupuncture Point. Evid Based Complement Alternat Med 2012;2012:781460. 23049611 10.1155/2012/781460 PMC3462424

[22] Ma SX Li XY Sakurai T Evidence of enhanced non-enzymatic generation of nitric oxide on the skin surface of acupuncture points: an innovative approach in humans. Nitric Oxide 2007;17:60–8. 17613264 10.1016/j.niox.2007.05.004

[23] Zhang WB Tian YY Zhu ZX The distribution of transcutaneous CO2 emission and correlation with the points along the pericardium meridian. J Acupunct Meridian Stud 2009;2:197–201. 20633492 10.1016/S2005-2901(09)60055-8

[24] Beissner F Marzolff I Investigation of acupuncture sensation patterns under sensory deprivation using a geographic information system. Evid Based Complement Alternat Med 2012;2012:591304. 23243458 10.1155/2012/591304 PMC3518766

[25] Langevin HM Churchill DL Wu J Evidence of connective tissue involvement in acupuncture. FASEB J 2002;16:872–4. 11967233 10.1096/fj.01-0925fje

[26] Langevin HM Yandow JA Relationship of acupuncture points and meridians to connective tissue planes. Anat Rec 2002;269:257–65. 12467083 10.1002/ar.10185

[27] Gao XY Li YH Liu K Acupuncture-like stimulation at auricular point Heart evokes cardiovascular inhibition via activating the cardiac-related neurons in the nucleus tractus solitarius. Brain Res 2011;1397:19–27. 21596372 10.1016/j.brainres.2011.04.034

[28] Minagawa M Kurono Y Ishigami T Site-specific organ-selective effect of epifascial acupuncture on cardiac and gastric autonomic functions. Auton Neurosci 2013;179:151–4. 23557872 10.1016/j.autneu.2013.03.005

[29] Cho ZH Chung SC Jones JP New findings of the correlation between acupoints and corresponding brain cortices using functional MRI. Proc Natl Acad Sci U S A 1998;95:2670–3. 9482945 10.1073/pnas.95.5.2670 PMC19456

[30] Hsiu H Hsu WC Hsu CL Assessing the effects of acupuncture by comparing needling the hegu acupoint and needling nearby nonacupoints by spectral analysis of microcirculatory laser Doppler signals. Evid Based Complement Alternat Med 2011;2011:435928. 21804856 10.1093/ecam/neq073 PMC3136473

[31] Wang G-j Tian Y-y Jia S-y Laterality and coherence analysis of Laser Doppler Flowmetry signals in bilateral Nèi guān (PC 6): a potential non-invasive method to assess microdrculatory changes of people in different ages. World J Acupunct Moxibustion 2017;27:47–52.

[32] Hsieh CL Chang YM Tang NY Time course of changes in nail fold microcirculation induced by acupuncture stimulation at the Waiguan acupoints. Am J Chin Med 2006;34:777–85. 17080544 10.1142/S0192415X06004284

[33] Zhang WB Wang YP Li HY Analysis on correlation between meridians and viscera in book the Yellow Emperor's Internal Classic. Zhen Ci Yan Jiu 2018;43:424–9. 30094978 10.13702/j.1000-0607.180185

[34] Liu WT Zhang LP Zheng MF Current researches and ideas regarding correlation between meridians and viscera. Zhen Ci Yan Jiu 2018;43:430–2. 30094979 10.13702/j.1000-0607.180069

[35] Kong J Kaptchuk TJ Webb JM Functional neuroanatomical investigation of vision-related acupuncture point specificity--a multisession fMRI study. Hum Brain Mapp 2009;30:38–46. 17990299 10.1002/hbm.20481 PMC4009985

[36] Fan H Zhao L Cui J Effects of acupuncture at acupoints on heart rate variability in patients with chronic stable angina pectoris. Chin J Tradit Chin Med Pharm 2017;32:1798–803.

[37] Rong P Zhu B Li Y Mechanism of acupuncture regulating visceral sensation and mobility. Front Med 2011;5:151–6. 21695619 10.1007/s11684-011-0129-7

